# Activity of Selected Antioxidant Enzymes, Selenium Content and Fatty Acid Composition in the Liver of the Brown Hare (*Lepus europaeus* L.) in Relation to the Season of the Year

**DOI:** 10.1007/s12011-015-0385-5

**Published:** 2015-06-05

**Authors:** Radosław Drozd, Renata Pilarczyk, Bogumiła Pilarczyk, Arleta Drozd, Agnieszka Tomza-Marciniak, Teresa Bombik, Małgorzata Bąkowska, Elżbieta Bombik, Dorota Jankowiak, Agata Wasak

**Affiliations:** Department of Immunology, Microbiology and Physiological Chemistry, West Pomeranian University of Technology in Szczecin, Aleja Piastów 45, 70-311 Szczecin, Poland; Laboratory of Biostatistics, West Pomeranian University of Technology in Szczecin, Judyma 10 Street, 71-466 Szczecin, Poland; Department of Animal Reproduction Biotechnology and Environmental Hygiene, West Pomeranian University of Technology in Szczecin, Judyma 6 Street, 71-466 Szczecin, Poland; Department of Biochemistry and Human Nutrition, Pomeranian Medical University, Broniewskiego 24, 71-460 Szczecin, Poland; Department of Reproduction and Animal Hygiene, University of Life Sciences, 20-950 Siedlce, Poland

**Keywords:** Brown hare (*Lepus europaeus L.*), Selenium, Fatty acids, Antioxidative enzymes

## Abstract

The aim of the study was to evaluate the effect of low concentrations of selenium in the environment on the activity of selected antioxidant enzymes: Se-GSHPx, total GSHPx, SOD, CAT, and GST as well as fatty acid profile in the livers of brown hares during winter and spring. Liver tissues obtained from 20 brown hares collected in the north-eastern Poland in the winter and spring season were analyzed. In the tissue analyzed, a significantly lower level of selenium was noticeable in the spring compared to winter; however, values measured in both seasons indicated a deficiency of this element in the analyzed population of brown hares. There were no differences found that could indicate the influence of Se deficiency on the activity of antioxidant enzymes. The determined activity of antioxidant enzymes and fatty acid composition suggest a negligible impact of the low concentration of Se on the analyzed biochemical parameters of brown hare livers.

## Introduction

A significant decline in the population of brown hares has been observed in the last 30 years in many European countries. The brown hare population in Poland has decreased by approximately 55 %. One of the reasons for this process is environmental changes. Development of communication infrastructure, urbanization of areas (which until now had been the refuge of brown hare), increased application of chemicals in agriculture, and establishment of large farms have negatively affected population size of these animals [1, 2]. Furthermore, a rapidly growing population of red foxes (*Vulpes vulpes* L.), that is the result of the success of the aerial rabies vaccinations and less interest in hunting, is also a critical factor influenced on decreasing the brown hare population [3]. The occurrence of brown hares is closely linked with an agricultural landscape, as they are significantly less common in forested areas [4, 5]. In addition to human and predators pressure, brown hares as wildlife animals are also subjected to nutritional stress associated with seasonal changes that affect the availability and quality of food, which is reflected in the rate and type of metabolism in the body of the animal [6, 7].

Oxidative stress (OS) is one of the consequences of seasonal changes in the environment, and it is utilized to determine the state of imbalance in the quantity of reactive oxygen species (ROS) and their derivatives [8, 9]. ROS excess leads to the formation of toxic metabolites, and thus damage to cellular organelles. Reactive oxygen species affect cellular lipid metabolism leading to their peroxidation and changes in the properties of cell membranes [10, 11]. Unbalanced ROS levels also lead to the damage of nucleic acids and proteins [12, 13].

The liver, due to its function in the body, is particularly exposed to the adverse effects of ROS and other oxidants. The proper functioning of this organ allows adapting to the changing conditions of the external environment and maintaining homeostasis of the entire organism. Liver cells are equipped with a specialized enzymatic system, which is responsible for maintaining an optimal level of ROS. On the first line of enzymatic antioxidants, defense is situated in an “enzyme triad,” which is formed by three main enzymes, superoxide dismutase (SOD), catalase (CAT), and glutathione peroxidase (GSHPx) [14, 15]. Additionally, the “triad” is supported by many other enzymes of which glutathione S-transferase (GST) activity can be used by cells in case of deficiency of selenium-dependent glutathione peroxidase activity (Se-GSHPx) [16]. GST plays also an important role in the second phase of detoxification of foreign compounds in cells [17]. The liver is the major organ responsible for selenium homeostasis in the body [18–20]. Selenium stored in hepatocytes is used inter alia for the synthesis of Se-GSHPx and other enzymatic selenoproteins. For this reason, selenium deficiency can affect the activity of certain Se-dependent enzymes and directly influence the fatty acid profile in liver cells [21–24]. Therefore, this is one of the reasons for paying more attention in recent years to the health consequences associated with a deficiency of this trace element. This is an issue of such importance, because a large part of Europe, including some regions of Poland, is considered as areas poor in selenium [25–27]. Issues of oxidative stress and changes in antioxidant enzyme activity associated with a sequence of seasons (changes in the physical conditions of the environment and access to food and its quality) and metabolism of fatty acids is weakly understood in the brown hare. With this in mind, we decided to examine the following hypothesis; the existence of brown hares on potentially Se deficient areas has an important impact on the activity antioxidant enzymes and fatty acid profiles in their liver.

The aim of this study was to compare the activity of selected antioxidant enzymes (SOD, CAT, GST, total GSHPx, and Se-GSHPx), selenium content, and profile of fatty acids in the brown hare liver, depending on the season.

## Materials and Methods

### Materials

The material for the study (livers) was collected from 20 brown hares (*Lepus europaeus L.*) in the spring (*n* = 10) and winter (*n* = 10). Animals were obtained by hunters within the established hunting limits in the region of Siedlce (52°10′00″N 22°16′30″E) during the hunting season in the years 2011–2012. The animals collected in the spring were a group of brown hares that have died as a result of conducting field work carried out by farmers. Material was stored at −80 °C until chemical analysis.

### Methods

#### Determination of Selenium Concentration

Selenium concentrations were determined using the spectrofluorometric method [28]. The samples (0.5–1.5 g) were digested in HNO_3_ at 230 °C for 180 min and in HClO_4_ at 310 °C for 20 min. Then 3 ml 9 % HCl was added to reduce Se^6+^ to Se^4+^. Selenium was derivatized with 2,3-diaminonaphthalene (Sigma-Aldrich), under controlled pH conditions (pH 1–2), with the formation of selenodiazole complex. This complex was extracted into cyclohexane. EDTA and hydroxylamine hydrochlorine were used as masking agents. Se concentration was determined fluorometrically using a Shimadzu RF-5001 PC spectrofluorophotometer and expressed in mg kg^−1^ of wet weight. The excitation wavelength was 376 nm, and the fluorescence emission wavelength was 518 nm. All chemicals used were of analytical reagent grade. The accuracy of the method was verified using the certified reference material BCR-185R (bovine liver) with Se concentration 1.68 mg kg^−1^ ww. A reference sample was analyzed in triplicate. The mean Se concentration was 95.8 ± 3.3 % of the reference values. The detection limit was 0.004 mg kg^−1^ wet weight.

#### Analysis of Fatty Acids

Fatty acids were extracted according to Folch et al. [29]. Liver tissue was powdered in liquid nitrogen and saponified with 1 ml of 2 M KOH methanol solution at 70 °C for 20 min and then methylated with 2 ml 14 % solution of boron trifluoride in methanol under the same conditions. In the next step, 2 ml of *n*-hexan and 10 ml saturated NaCl solution were added. Samples were positioned until the upper (*n*-hexan phase) and lower layers were completely separated. One milliliter of the *n*-hexan phase was collected.

##### Gas Chromatography Analysis

Gas chromatography was performed using an Agilent Technologies 7890A GC System. The instrument was equipped with a SUPELCOWAX™ 10 Capillary GC Column (L × I.D. 15 m × 0.10 mm, df 0.10 μm) (Supelco, Cat No. 24343). Chromatographic conditions were as follows: the starting temperature was 60 °C for 0 min, increased at a rate of 40 °C/min to 160 °C for 0 min, next increased at a rate of 30 °C/min to 190 °C for 0.5 min, and subsequently increased at a rate of 30 °C/min to 230 °C for 2.6 min, at which it was maintained for 4.9 min. The total time of analysis was 8 min and the gas flow rate was 0.8 ml/min with nitrogen as the carrier gas. Identification of fatty acid methyl esters in the samples was carried out by comparing their retention time to standards. The fatty acids were summed according to the following equations: saturated fatty acid (SFA) = C14 : 0 + C15 : 0 + C16 : 0 + C18 : 0; monounsaturated fatty acid (MUFA) = C16 : 1 + C18 : 1 + C18 : 1n − 9 + C20; polyunsaturated fatty acid (PUFA) = 18 : 2n − 6 + 18 : 3n − 3 + 20 : 4n − 6 + 20 : 5n − 3 + 22 : 6n − 3.

#### Antioxidant Enzyme Assays

##### Sample Preparation

Samples of liver tissue were homogenized in a mechanical homogenizer on ice with 10 volumes of 50 mM Tris-Cl buffer pH 8.1, 1 mM PMSF, 2 mM EDTA. Homogenates were centrifuged for 15 min at 15,000 rpm at 4 °C. Supernatants were collected and stored until analysis at −80 °C. Total protein was assayed by Bradford method with bovine serum albumin as a standard [30].

##### Total GSHPx and Selenium-Dependent Glutathione Peroxidase Assay

Glutathione peroxidase activity was assayed according to Pagila and Valentine [31] using RANSEL kit (Randox Laboratories Ltd). Cumene hydroperoxide was used to determine the total activity of GSHPx as a substrate. Se-GSHPx was assayed with hydrogen peroxide as a substrate at a final concentration of 0.25 mM and the presence of 1 mM sodium azide. The activity was defined as the amount of total GSHPx or Se-GSHPx that oxidizes 1 nanomole of NADPH per min per milligram of extracted protein at 37 °C and pH 7.2.

##### Superoxide Dismutase Activity Assay

Superoxide dismutase activity was determined by its ability to inhibit the auto-oxidation of pyrogallol using the method of Marklund and Marklund [32] modified to microplate assay. Nine microliters of 10 μM bovine erythrocyte catalase solution in 50 mM Tris-Cl, pH 8.5, and 9 μl of 24 mM (in 10 mM HCl) pyrogallol solution was added to 270 μl of 50 mM Tris-Cl, 1 mM EDTA buffer pH 8.5. Subsequently, 6 μl of appropriately diluted samples were added to the reaction mixture. The increase in absorbance was monitored at 420 nm (Tecan, Infinite m200 PRO) after 2 min at 15-s intervals after a 30-s lag phase at 25 °C. One unit of SOD reduced auto-oxidation of pyrogallol to 50 % at pH 8.5 at 25 °C.

##### Catalase Activity Assay

Catalase activity was determined according to Li and Schellhorn [33]. Ten microliters of the sample diluted in assay buffer (50 mM phosphate buffer pH 7.0) were added to 300 μl of 5 mM hydrogen peroxide solution in assay buffer, brought to 25 °C in flat-bottomed 96-well UV-transparent microtitre plate (Greiner Bio-One, GmbH). Immediately after sample addition, the decrease in absorbance was monitored at 240 nm for 2 min at 15-s intervals (Tecan, Infinite m200 PRO). One unit of catalase broke down one micromole of hydrogen peroxide to oxygen and water per min, at pH 7.0 at 25 °C.

##### Glutathione S-Transferase Activity Assay

Twenty microliters of diluted (0.1 M phosphate buffer, 2 mM EDTA, pH 6.5) buffer sample were transferred to 180 μl of assay buffer with 1-mM reduced glutathione and 2-mM 1-Chloro-2,4-dinitrobenzene [34]. After 1 min of lag time, absorbance was measured for 4 min at 15-s intervals at 25 °C. One unit of GST-specific activity is defined as the amount of enzyme that catalyzes the formation of 1 μmol of product per min per milligram of the extracted protein at pH 6.5 at 25 °C .

### Statistical Analysis

Statistical calculations were performed using STATISTICA PL 9.0 software. All data are expressed as an arithmetic mean and standard error of the mean (SE). Fatty acid composition, enzyme activities, and Se concentrations were log-transformed to attain or approach a normal distribution of the data. The significance of differences between the seasons was tested with Student’s *t* test. Differences were considered significant at *p* < 0.05 and *p* < 0.01. Relationships between the activity of enzymes and fatty acid composition as well as relationships between concentrations of selenium in the liver and fatty acid composition were assessed by calculating correlation coefficients. Pearson’s correlation coefficient (*r*_*x*,*y*_) was calculated for each season separately and for all data combined. Statistical significance of correlation coefficients was tested at *p* < 0.05 and *p* < 0.01.

## Results

### Activity of Antioxidant Enzymes and Selenium Level

The average activity of total GSHPx and Se-GSHPx, in both seasons studied (spring, winter), was at a similar level. The activity of Se-GSHPx in both seasons represented 57 % of the total activity of GSHPx. The analyzed CAT and GST activities did not reveal any significant differences between the seasons. In the winter season, a lower level of SOD activity compared to spring was observed; however, this difference was not statistically significant. The average concentration of selenium in the liver of brown hares in winter was significantly (*p* < 0.001) higher compared to the spring season (Table 1).

**Table 1:** Average values and comparison of antioxidant enzymes activity and selenium content in the analyzed livers of brown hares depending on seasons

	Season				
	Spring (*n* = 10)	SE	Winter (*n* = 10)	SE	*p* value
Total GSHPx (U/mg)	370.4	50.8	371.7	68.0	0.73
Se-GSHPx (U/mg)	212.1	27.8	221.0	57.4	0.14
CAT (U/mg)	40.2	6.6	38.8	9.25	0.90
SOD (U/mg)	33.1	2.9	24.7	3.68	0.09
GST (U/mg)	1.2	0.1	1.4	0.29	0.49
Se (mg kg^−1^ ww)	0.063^A^	0.0031	0.099^A^	0.0058	<0.001

The same uppercase letters denote statistically significant differences at *p* < 0.001

### Fatty Acid Composition in the Liver Tissue of Brown Hares

Fatty acid profile of brown hare’s livers did not differ significantly between seasons (Table 2). The highest content of polyunsaturated linoleic acid (C18:2*n-6*) was found in both seasons, followed by saturated stearic (C18:0) and palmitic (C16:0) acids. Oleic acid was dominant (C18:1*n-9*) in the group of monounsaturated fatty acids. The total content of polyunsaturated fatty acids (PUFA) was high and amounted to over 43 %, similarly as the total content of saturated fatty acids (SFA, 42–43 %). The ratios of PUFA/SFA and *n-6*/*n-3* PUFA were favorable and amounted to 1.0 and 1.7, respectively.

**Table 2:** Fatty acid composition (%) in the analyzed livers of hares depending on seasons

Fatty acid	Seasons				
	Spring (*n* = 10)		Winter (*n* = 10)		
	%	SE	%	SE	*p* value
C14:0 (myristate)	0.7	0.13	0.7	0.14	ns
C15:0 (pentadecanoate)	0.3	0.04	0.3	0.02	ns
C16:0 (palmitate)	17.9	0.54	19.7	1.27	ns
C18:0 (stearate)	23.1	1.12	22.3	1.71	ns
C16:1 (palmitoleate)	1.0	0.10	1.0	0.14	ns
C18:1 (trans vaccenate)	2.5	0.17	2.5	0.17	ns
C18:1*n*-9 (oleate)	10.8	1.19	9.6	1.16	ns
C18:2*n*-6 (linoleate)	26.2	1.75	26.2	1.30	ns
C18:3*n*-6 (gamma linoleate)	0.5	0.06	0.3	0.06	ns
C18:3*n*-3 (linolenate)	7.0	0.90	8.8	1.27	ns
C18:4*n*-3 (stearidonate)	0.3	0.06	0.2	0.02	ns
C20:1 (eicosenate)	0.2	0.04	0.1	0.05	ns
C20:3*n*-3 (eicosatrienoate)	5.2	0.76	4.7	0.47	ns
C20:4*n*-6 (arachidonate)	0.2	0.05	0.1	0.05	ns
C20:5*n*-3 (eicosapentanoate)	1.2	0.23	1.1	0.27	ns
C22:4*n*-6 (docosatetraenoate)	0.3	0.07	0.6	0.21	ns
C22:5*n*-3 (docosapentaenate)	1.7	0.49	1.2	0.13	ns
C22:6*n*-3 (docosahexanoate)	1.0	0.24	0.6	0.08	ns
ΣSFA	42.0	0.80	43.1	0.75	ns
ΣMUFA	14.5	1.24	13.1	1.18	ns
ΣPUFA	43.5	1.52	43.8	1.02	ns
ΣUFA^a^	58.0	0.80	56.9	0.75	ns
Σ*n-3* PUFA	16.1	2.72	16.4	3.5	ns
Σ*n-6* PUFA	27.2	12.9	27.2	12.9	ns
PUFA/SFA	1.04	0.049	1.02	0.031	ns
MUFA/SFA	0.35	0.029	0.31	0.031	ns
UFA/SFA	1.39	0.047	1.33	0.040	ns
*n-6*/*n-3* PUFA	1.78	0.231	1.75	0.203	ns

^a^ *UFA* Unsaturated fatty acid—sum of MUFA and PUFA

Analysis of correlation between enzyme activity and the concentration of fatty acids is shown in Table 3. In the spring, significant (*p* ≤ 0.05) positive correlations were demonstrated between the activity of total GSHPx and the content of C18:0 acid, as well as the activity of total GSHPx and saturated fatty acids. The CAT activity was positively correlated with the content of C15:0, C20:3*n-3*, and C22:5*n-3* fatty acids, while the SOD activity was positively correlated with the content of C18:4 acid and the total content of monounsaturated fatty acids (MUFA). Negative correlations were found between the activity of total GSHPx and the content of C22:5*n-3* acid, while significant negative correlations were observed between the CAT activity and the C18:0 acid content, the SOD activity and the C18:1*n-9* acid content, and the SOD activity and the total content of polyunsaturated fatty acids. In addition, there was a negative correlation between the activity of GST and the content of C22:4 acid (Table 3).

**Table 3:** Pearson’s correlation coefficient between the activity of antioxidant enzymes and fatty acid content in different seasons in the liver of examined hares

Value of the coefficient of correlation (*r*_*x.y*_) between:	Season	
	Spring	Winter
Total GSHPx - C16:0	–	0.82*
Total GSHPx - C18:0	0.68*	–
Total GSHPx - C18:1*n*-9	–	0.82*
Total GSHPx - C18:1	–	−0.91**
Total GSHPx - C20:3*n*-3	–	−0.84*
Total GSHPx - C22:6*n*-3	−0.66*	−0.75*
Total GSHPx - SFA	0.64*	
Total GSHPx - MUFA	–	0.77*
Total GSHPx - PUFA	–	−0.92**
CAT - C15:0	0,71*	–
CAT - C18:0	−0.68*	–
CAT - C20:3	0.70*	0.85*
CAT - C22:5*n*-3	0.70*	–
SOD - C18:1*n*-9	−0.71*	–
SOD - C18:4	0.74*	–
SOD - MUFA	0.78**	–
SOD - PUFA	−0.71*	–
GST - C18:0	0.70*	–
GST - C22:4	−0.71*	–

*p < 0.05 (statistically significant coefficient of correlation); **p < 0.01 (statistically significant coefficient of correlation)

In the winter, significant (*p* < 0.05), very high positive correlations were observed between the total GSHPx activity and the content of C16:0 acid, C18:1*n-9* acid, and the total amount of monounsaturated fatty acids. A positive correlation was also found between the CAT activity and the C20:3*n-3* acid content. Significant negative correlations were recorded between the total GSHPx activity and the content of C18:1, C20:3*n-3* and C22:6*n-3*, and PUFA.

There was no significant correlation between the concentration of selenium in the liver and the content of individual fatty acids and antioxidant enzyme activities.

## Discussion

Seasonal environmental changes tend to have a significant impact on many biochemical parameters of the liver. Temperature changes and food availability, which are a limiting factor for the general condition of the organism, are often reflected in the content of trace elements in that organ, which translates into the activity of particular antioxidant enzymes that affect the qualitative composition of fatty acids in the phospholipids of cell membranes.

Referring Se concentrations determined in the liver of brown hares to the biochemical criteria used in the diagnosis of selenium deficiency in the liver of rabbit (below 0.4 mg kg^−1^ ww—deficiency; 0.60–1.00 mg kg^−1^ ww—marginal level; 1.07–2.00 mg kg^−1^ ww—optimal level) [35], selenium deficiency has been shown in all of the subjects. Similar results in the analyzed area were obtained by Dębski et al. [36]. Although these authors also demonstrated an insufficient level of this element (0.255 mg kg^−1^ ww) in brown hares, it was significantly higher compared to the results obtained in the present study. Linšak et al. [37] reported selenium deficiency in brown hares in Croatia, from the cadmium unpolluted area (0.332 mg kg^−1^ ww) and contaminated areas (0.153 mg kg^−1^ ww).

The content of selenium in plants varies, depending both on the soil richness in this element as well as chemical form and plant species that are critical for its availability for animals [38]. Plants absorb selenium predominantly in the form of selenate (SeVI) and selenite (SeIV) or sometimes in the form of selenides, depending on the plant species. Translocation of selenium from the roots to the shoots is primarily dependent on the chemical form. Selenate (SeVI) is more easily transported than selenite (SeIV). This is due to a faster transformation of selenate into organic forms retained in the roots. Therefore, the translocation to the stems of the plants is limited. It has been shown that selenate is more mobile in the xylem and only small amounts are converted into the organic form of this trace element [39, 40]. The higher concentration of selenium in the examined livers was found during the winter, when brown hares potentially have difficulties in maintaining a varied diet.

In this period, brown hares can eat roots and other underground parts of plants and bark of trees (apple, willow, hawthorn, poplar, black locust, and oak), which are richer in selenium [6]. In the winter, European hare often also feed on available winter crops like winter wheat [41]. In this season, the elevated level of selenium can be liberated to the atmosphere from coal and oil combustion as result of low-emission, which sources are small heating plants or a single-family home. According to Yudovich and Ketris [42] Se contents (on an ash basis) in hard coals and brown coals are 9.9 ± 0.7 and 7.6 ± 0.76 mg kg^−1^, respectively. In the atmosphere, Se is transformed to many forms and finally with the rainwater can be transferred to soil and absorbed by plants [43]. The periodical increasing in the atmospheric concentration of Se is the factor that probably has also influence on seasonal increasing in selenium content in the available for brown hares plants and observed changes in its concentration in their liver between the seasons.

Due to the critical function of Se in the structure of selenium-dependent glutathione peroxidase, it can be expected that the deficiency of Se in both seasons will be evidenced by the reduced activity of this enzyme. However, there was no considerable difference in the activity of Se-GSHPx in brown hare livers, despite a significantly higher selenium level in the winter season. Both in winter and spring, the content of this trace element was at a level indicative of its extreme deficiency. In a close relative of the brown hare, the rabbit (*Oryctolagus cuniculus*), the lack of decrease in the Se-GSHPx activity was shown in the liver of animals fed with feed devoid of selenium [44–46], whereas Muller et al. [47] demonstrated in rabbit liver a statistically significant decrease in Se-GSHPx activity under selenium deficiency, however, not at a considerable level, compared to the heart, kidney, and *musculus longissimus dorsi*. The decrease in the selenium-dependent activity of glutathione peroxidase was also observed in other animals, such as chickens, cows, and pigs [48–50]. Liver cells express both Se-GSHPx and non-Se-GSHPx. Arthur et al. [16] have shown in rat hepatocytes that Se-GSHPx activity was decreased, while that of GST was significantly increased under conditions of selenium deficiency. The nature of brown hare, that are extremely fast runners, has probably forced a development in these animals an appropriate metabolic pathways, that are efficient even in case of deficiency of such trace elements as selenium. During the extended run, how it was observed in young and old rats, a large amount of ROS is created. The consequence of this is a reduction in endogenous antioxidant level and increasing of lipid peroxidation in liver [51]. As reported by Sun et al. [52], the endurance exercise for rats causes increasing GST activity in liver with no significant elevation of total GSHPx activity. Glutathione S-transferases in this case had increased peroxidase activity, associated with the changes of composition the enzyme subunit [53]. This observation shed some light on possible mechanism of resistance on low level of Se in liver of brown hare. The insufficiency of Se-GSHPx is probably compensated in brown hare livers by an increased activity of GST that serves as alternative protection against ROS [54–57]. Due to the lack of data regarding the GST activity level in brown hare livers, it is difficult to ascertain whether the determined level of activity of this enzyme differs from the reference values.

Selenium content may have a significant impact on the composition of fatty acids in the liver [58]. It was found that a deficiency of this element in rats resulted in a reduction of long-chain fatty acid contents, such as C20–C22, and a decrease of *n-3* quantity relative to *n-6* [23]. Despite statistically significant differences between winter and spring in the Se hepatic content of the examined brown hares, there were no such differences in the FA profile. This may be due to the fact that these animals lived in the same land space deficient in selenium. PUFA content in both seasons was at a level similar to that in the study of Valencak et al. [59] for *L. europaeus* and Benatmane et al. [60] and Li et al. [61] in rabbits. During the two seasons, the highest fraction in PUFA was linoleic acid (C18:2n-6). Valencak et al. [59] also showed that brown hares with a high content of this acid had low levels of arachidonic acid (C20:4*n-6*), which was confirmed in the current study. According to Popescu et al. [62], brown hares may generally show a preference for selecting plants with a high content of linoleic acid that results in the higher amount of this fatty acid in the liver. This is also true for other mammalian herbivores but the proportion of linoleic acid and its *n-6* derivates is even higher in brown hares than in other mammals of comparable body size that is related with their ability to very fast running [63, 64]. Vitamin E, in addition to selenium, is an important factor influencing the profile of fatty acids [65, 47]. Under the conditions of Se deficiency, it is often primarily responsible for maintaining the integrity of cell membranes and inhibition of progressive lipid peroxidation [66, 67]. In the spring, brown hares feed on crops, such as growing corn, weeds, and various herbs, while they nibble at the branches of trees and shrubs as well as the bark of thicker branches and tree trunks in the winter [68, 41]. The food available in spring probably provides higher amounts of vitamin E that can compensate greater than in the winter selenium deficiency.

Availability and variety of food in habitat of the examined brown hares, which depends on the season, seems to be the most important factor that affects the level of selenium in their livers. The obtained results may indicate priority in the distribution of Se to the synthesis of selenium-dependent glutathione peroxidase even in the case of a strong deficiency of this trace element. A high content of unsaturated fatty acid may indicate the maintaining of redox homeostasis in brown hare liver despite a very low selenium level. The deficiency of this trace element was probably compensated by the mobilization of the alternative endogenous antioxidant defense mechanisms.
